# Findings From Implementation of the EIT-4-BPSD Trial

**DOI:** 10.1093/geroni/igab046.588

**Published:** 2021-12-17

**Authors:** Barbara Resnick, Marie Boltz, Ann Kolanowski

## Abstract

Behavioral and psychological symptoms of dementia (BPSD) include aggression, agitation, depression, anxiety, apathy and hallucinations and are exhibited by up to 90% of nursing home (NH) residents with dementia. BPSD result in negative health outcomes, functional decline, high care costs, increased risk for inappropriate use of antipsychotic medications and social isolation. Behavioral approaches are endorsed as the first line of treatment for BPSD. Despite regulatory requirements, less than 2% of nursing homes consistently implement these approaches. The EIT-4-BPSD Trial was done to test a novel implementation approach to assure that staff in nursing homes use non-pharmacologic, behavioral approaches for the management of BPSD. EIT-4-BPSD is a theoretically-based four-step approach that includes: 1. Assessment of the environment and policies; 2. Education of staff; 3. Establishing person-centered care plans; and 4. Mentoring and motivating staff. Implementation of the four-step approach was guided by the Evidence Integration Triangle (EIT). The EIT brings together evidence and key stakeholders from the NH and uses a participatory implementation processes, practical evidence-based interventions, and pragmatic measures of progress toward goals. A total of 55 nursing homes from two states and 553 residents were included in this study. Nursing homes were randomized to EIT-4-BPSD or education only. This symposium will describe the utility of the EIT as an implementation framework based on the Reach, Effectiveness, Adoption, Implementation, and Maintenance model, report detailed effectiveness outcomes of EIT-4-BPSD at the setting and resident levels, and address recruitment and measurement challenges and future solutions to these challenges.

